# Significance of Inflammation Markers to Predict Curative Treatment for Prostate Cancer Patients on Active Surveillance

**DOI:** 10.1002/jcla.70059

**Published:** 2025-05-31

**Authors:** Yenigürbüz Serkan, Ediz Caner, Akan Serkan, Kati Bulent, Vural Yasin, Alcin Adem, Yilmaz Omer

**Affiliations:** ^1^ Department of Urology Sultan 2. Abdulhamid Han Education and Research Hospital Istanbul Turkey; ^2^ Department of Urology Fatih Sultan Mehmet Han Education and Research Hospital Istanbul Turkey; ^3^ Department of Urology Harran University Hospital Sanliurfa Turkey

**Keywords:** active surveillance, biomarkers, inflammation mediators, prostatic neoplasms, tumor

## Abstract

**Purpose:**

Active surveillance (AS) strategy aims to avoid unnecessary or excessive early treatment in patients at a low risk for prostate cancer (PCa). However, a biomarker that can predict the need for early curative treatment in patients under AS has not been identified to date. In this study, we aimed to investigate the potential of inflammatory biomarkers in predicting the requirement of curative treatment in the early period in patients under AS.

**Materials and Methods:**

This study included a total of 83 patients with the diagnosis of PCa and under AS. Patient age, prostate‐specific antigen (PSA) level, prostate volume (PV), PSA density (PSAD), neutrophil/lymphocyte ratio (NLR), platelet/lymphocyte ratio (PLR), systemic immune‐inflammation index (SII) and follow‐up period were compared between the groups.

**Results:**

There was a significant difference between the two groups in terms of PSAD, NLR, PLR and SII (*p* = 0.037, *p* = 0.046, *p* = 0.008, *p* = 0.004 and *p* = 0.005, respectively). The cut‐off value determined by performing ROC analysis to evaluate the levels that predict the need for curative treatment before AS was 0.125 for PSAD (sensitivity: 61.8%, specificity: 61.2%), 2.01 for NLR (sensitivity: 67.6%, specificity: 55.1%), 115.49 for PLR (sensitivity: 73.5%, specificity: 59.2%) and 465.40 for SII (sensitivity: 70.6%, specificity: 59.2%).

**Conclusions:**

The analysis of PSAD, NLR, PLR and SII before making the decision to conduct AS can guide clinicians regarding curative treatment in the early period.

## Graphical abstract

There was no difference between the groups in terms of analyses of histopathological parameters performed in testis tissue. While testicular volume, sperm motility and the ratio of sperm cells with normal morphology were relatively smaller before the level of the internal inguinal ring in the testicular descent, we could not find significant differences in the histopathological parameters between groups.

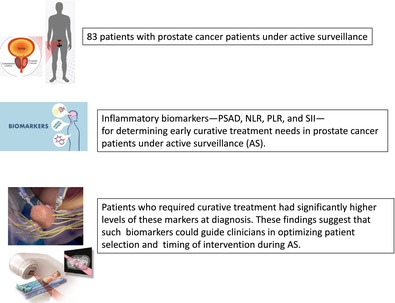

## 1 Introduction

Prostate cancer (PCa) is the second most commonly diagnosed cancer in men. Moreover, 1.6 million men are diagnosed with PCa, and 366,000 men die each year [1]. Increased population screening for PCa in recent years has led to a reduction in advanced disease and disease‐specific mortality. However, early diagnosis and treatment increase the possibility of side effects during disease management at an early age [2, 3]. These issues are extremely important in PCa screening and treatment policies because overdiagnosis during PCa screening is often considered the most important reason that can lead to potential harm [4]. In the future, patient selection and continued advancements of biomarkers and clinical staging should focus on further reducing both overdiagnosis and overtreatment.

In light of these concerns, active surveillance (AS) is considered a safe and acceptable disease management strategy for low‐risk PCa. Some prospective studies in the literature have focused on the place and long‐term results of AS in PCa and reported that the probability of mortality in patients with PCa followed up under AS is very low [5, 6]. However, the most important concern associated with AS is that the possibility of curative treatment may be lost during disease management [3]. Close follow‐up and evaluation of patients by performing repeated biopsies focus on reducing this possibility. AS is terminated for patients in whom there is a disease upgrade; an increase in disease extent, stage, and progression; or based on patient preference. Predicting the need for curative treatment in the early phase of the disease can eliminate repetitive PCa follow‐ups, the need for confirmation biopsy and the additional costs and morbidity associated with biopsy; it also helps clinicians in making more appropriate treatment choices in the early period of managing this curable disease.

However, no biomarker has been identified that predicts the progression of patients under AS and the need for curative treatment in the early period. The lack of a biomarker that can be evaluated in patients under AS before performing the recommended routine confirmation biopsies is an important barrier in the early detection of the need for curative treatment. The importance of biomarkers has been emphasised in many studies [7, 8]. In the present study, we evaluated the efficacy of markers associated with inflammation in predicting the need for curative treatment in patients under AS in the early period.

## 2 Patients and Methods

The study included 113 patients who underwent transrectal ultrasound‐guided prostate biopsy due to abnormality in DRM and/or elevated prostate‐specific antigen (PSA) in hospital between January 2015 and December 2020, were diagnosed with PCa based on pathological evaluation, and determined to be suitable for AS that is consistent with low‐risk PCa reported by the European Association of Urology.

**FIGURE 1:**
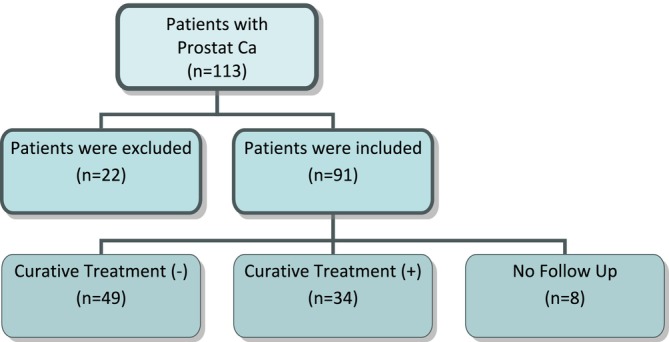
Flow chart of patients included and not included in the study.

Patients who did not prefer the AS treatment protocol, were treated for active infection before biopsy, whose hemogram evaluation could not be determined before biopsy, and all data could not be accessed were excluded from the study. The study was conducted with 83 patients who were compatible with active surveillance as disease management (Figure 1).

### 2.1 Study Design

The patients were divided into two groups in accordance with the need for curative treatment during AS. Patients who did not need curative treatment during AS were included in Group 1 (*n* = 49), whereas patients who needed curative treatment and therefore AS was terminated were included in Group 2 (*n* = 34).

Age, PSA level, prostate volume (PV), PSA density (PSAD=PSA/PV), Neutrophil/lymphocyte ratio (NLR), platelet/lymphocyte ratio (PLR) and systemic immune‐inflammation index (SII) were analyzed between the groups.

### 2.2 Statistical Analysis

Clinicopathological data were input to SPSS version 20 as categorical variables (digital rectal examination findings, pathological staging, Gleason grade) and continuous variables (age, PSA, PSAD, NLR, PLR, SII values and follow‐up period). Conformity of the data to normal distribution was checked by the Kolmogorov–Smirnov test. In addition to descriptive statistical methods (mean, standard deviation, frequency), normally distributed quantitative parameters were compared with the Student *t*‐test, whereas non‐normally distributed quantitative parameters were compared with the Mann–Whitney *U* test. Chi squared and Fisher's exact tests were used for comparing qualitative data. The optimal cut‐off point was determined with ROC curve analysis. Areas under the ROC curve in paired sequences were compared using the *z* equation. After ROC analysis, PPV and NPV values of the cut‐off values were calculated manually. All statistical analyses were performed in SPSS; a *p*‐value of < 0.05 was considered statistically significant in all analyses.

## 3 Results

The mean age of the patients participating in the study was 66.34 ± 7.75 years. The mean age of the patients was 64.97 ± 7.67 and 68.58 ± 7.52 years in Groups 1 and 2, respectively, and the difference was statistically significant (*p* = 0.037). DRM findings were found to be abnormal in 22.9% of the patients in Group 1 and 20.6% of the patients in Group 2, and there was no significant difference between the two groups (*p* = 0.802). All the patients belonged to grade 1 as per the International Society of Urologic Pathologists' grading system. Of the 83 patients, confirmation biopsy was performed in 73 (87.95%), and 12–24 core samples were collected at the 12th month of their follow‐up. Due to the decrease in PSA levels before confirmation biopsy and the detection of Prostate Imaging‐Reporting and Data System 1–2 lesions in multiparametric prostate magnetic resonance imaging findings, confirmation biopsy was not performed in 10 patients.

The statistical comparisons of the patients in Groups 1 and 2 in terms of PSA, PV, PSAD, NLR, PLR and SII at the time of diagnosis are summarised in Table 1. The median PSAD, NLR, PLR and SII were higher in Group 2, and the difference between the groups was statistically significant (*p* < 0.05).

**TABLE 1:** Analysis and statistical comparison of patients in Groups 1 and 2 in terms of available parameters.

Variables	Group 1, mean ± SD or median (IQR)	Group 2, mean ± SD or median (IQR)	*p*
Age (years)	63.92 ± 5.79 (57–74)	62.76 ± 5.84 (47–74)	0.037
Prostate volume (mL)	50 (27.5)	46 (30.75)	0.335
PSA (ng/mL)	6.01 (2.40)	7.64 (3.43)	0.051
PSAD (ng/mL/cc)	0.11 (0.05)	0.145 (0.10)	0.046
NLR	1.90 (0.90)	2.37 (1.60)	0.008
PLR	103.41 (68.55)	133.61 (76.82)	0.004
SII	433.07 (292.92)	714.18 (621.77)	0.005

**FIGURE 2:**
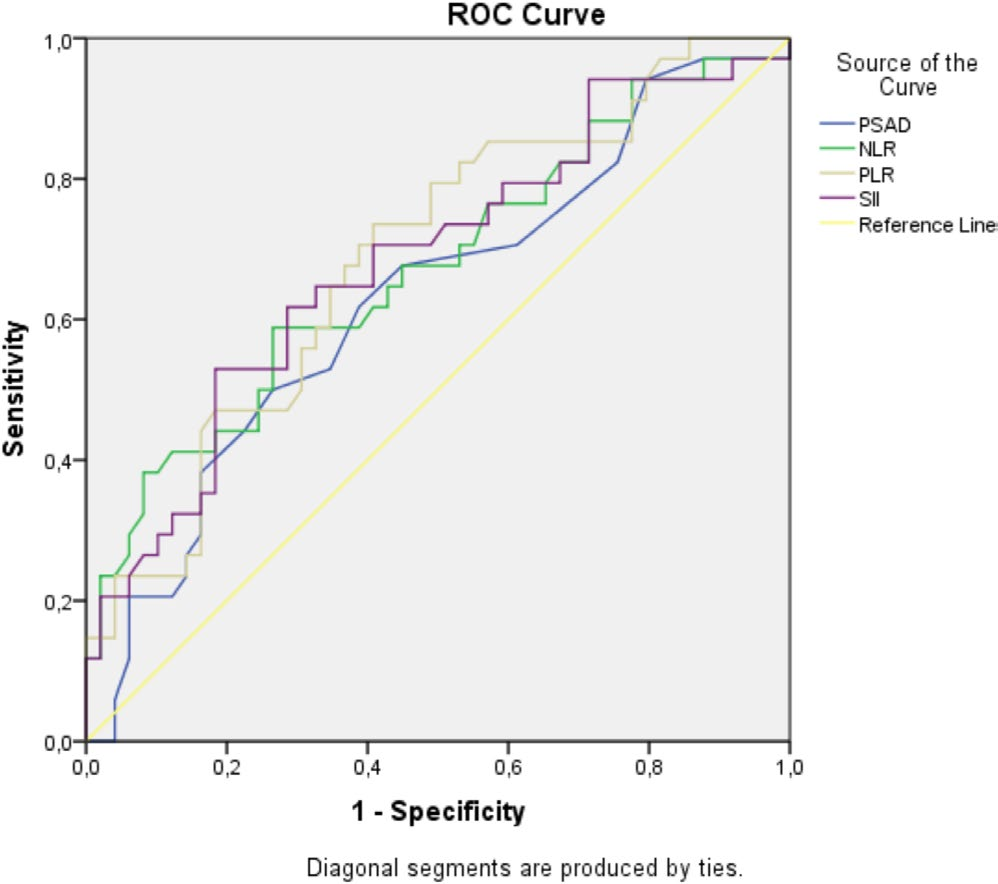
ROC graph of PSAD, NLR, PLR and SII to evaluate their efficacy in predicting the need for curative treatment in patients with PCa under AS.

A ROC curve outcome was reported for PSAD, NLR, PLR and SII in Figure 2. The results of the ROC analysis of PSAD, NLR, PLR, and SII for assessing their effectiveness in predicting the need for curative treatment in PCa patients under AS are shown in (Table 2).

A ROC curve was plotted for PSAD value to predict curative treatment requirement (Figure 2). Area under the curve (AUC) was 0.629 with a standard error of 0.05, significantly higher than 0.5 (*p* = 0.046). The cut‐off value of the PSAD for predicting curative treatment requirement was 0.125 with a sensitivity of 61.8% and a specificity of 61.2%. Positive predictive value (PPV) and negative predictive value (NPV) were 0.61 and 0.61, respectively.

A ROC curve was plotted for NLR value to predict curative treatment requirement (Figure 2). AUC was 0.673 with a standard error of 0.05, significantly higher than 0.5 (*p* = 0.008). The cut‐off value of the NLR for predicting curative treatment requirement was 2.015 with a sensitivity of 67.6% and a specificity of 55.1%. PPV and NPV were 0.55 and 0.68, respectively.

A ROC curve was plotted for PLR value to predict curative treatment requirement (Figure 2). AUC was 0.688 with a standard error of 0.05, significantly higher than 0.5 (*p* = 0.004). The cut‐off value of the PLR for predicting curative treatment requirement was 115.495 with a sensitivity of 73.5% and a specificity of 59.2%. PPV and NPV were 0.59 and 0.74, respectively.

A ROC curve was plotted for SII value to predict curative treatment requirement (Figure 2). AUC was 0.682 with a standard error of 0.05, significantly higher than 0.5 (*p* = 0.005). The cut‐off value of the SII for predicting curative treatment requirement was 465.4 with a sensitivity of 70.6% and a specificity of 59.2%. PPV and NPV were 0.59 and 0.71, respectively.

**TABLE 2:** ROC analysis results of PSAD, NLR, PLR and SII for evaluating their efficacy in predicting the need for curative treatment in patients with PCa under AS.

Area under the curve					
Test result variable(s)	Area	Std. error	Asymptotic sig.	Asymptotic 95% confidence interval	
				Lower bound	Upper bound
PSAD	0.629	0.063	0.046	0.506	0.752
NLR	0.673	0.061	0.008	0.553	0.794
PLR	0.688	0.059	0.004	0.573	0.804
SII	0.682	0.060	0.005	0.564	0.801

## 4 Discussion

The relationship between PCa and inflammatory changes has been revealed more frequently in recent years. In the present study, the efficacy of PSAD, NLR, PLR and SII evaluated before biopsy in patients followed up under AS in predicting the need for curative treatment in the early stages of disease management is clearly demonstrated. While some of the patients under AS have stable disease, some show progression [9]. In addition, there is always the possibility of overlooking high Gleason scores in repeat biopsy specimens during AS [10]. Therefore, the fact that PSAD, NLR, PLR and SII are relatively easy‐to‐evaluate parameters can be considered an advantage in determining the need for curative treatment.

NLR is known as an indicator of the immune system's response to various stress stimuli [11]. Tumour cells are also one of the stress stimuli inducing systemic inflammatory response [12]. In a systematic review of 100 studies, NLR was found to be associated with overall survival in many solid tumours [13]. Although the mechanism has not been clearly explained, the most widely accepted view is that increased neutrophil count activates inflammation‐induced angiogenesis and contributes to the nutrition and metastasis of tumour cells and decreased lymphocyte counts lead to a decrease in the amount of cytotoxic cytokines. Thus, increased NLR emerges as a poor prognostic factor [14, 15, 16] There are studies examining the relationship between PCa and NLR in the literature, but these studies predominantly focus on castration‐resistant metastatic PCa [17, 18, 19]. To a lesser extent, the relationship between NLR and overall survival after radiotherapy or radical prostatectomy in patients with localised PCa has also been investigated [20, 21, 22, 23]. Lee et al. determined the NLR threshold value as 2.5 and stated that NLR was positively correlated with Gleason score, pathological stage and extracapsular extension [24]. Zhang et al. reported similar results in another study; it was stated that a high NLR value (> 2.36) may be an indicator of an increase in the pathological stage and lymph node involvement [17]. Cao et al. reported that although prostate capsule invasion was higher in patients with high NLR, this finding was not statistically significant [11]. In contrast to these studies, Kwon et al. reported that NLR could not be used as a predictive marker in patients with low‐risk PCa [25]. Bahing et al. also reported a negative significant correlation between neutrophil count and overall survival in their large cohort study including localised PCa cases, and they could also not find a relationship between other inflammatory markers including NLR [26]. Kwon et al. showed that high NLR values in patients who underwent robot‐assisted radical prostatectomy after AS were associated with an increase in disease stage as a result of postoperative pathology [25]. In the present study, NLR was found to be higher in patients who underwent definitive treatment after AS termination (*p* = 0.008). There are studies in the literature that support the results of the present study. However, there are some studies showing that NLR is not significant in patients with low‐risk PCa, but it can be used as a predictive biomarker especially in the low‐risk patient group such as those under AS. Therefore, it is essential to determine the cut‐off value of NLR. In their study involving 2067 patients with PCa, Jang et al. determined the median NLR value to be 1.76 [25, 27]. Nuhn et al. and Minardi et al. reported a cut‐off value of 3 [23, 28]. In their study including 1137 patients under active follow‐up, Kwon et al. found the cut‐off value of NLR to be 2.6, which was also the median value [25]. In the statistical analysis performed in the present study, the cut‐off value of NLR was determined to be 2.015, which is lower compared to that of other studies in the literature. This may be attributed to the presence of a low degree of inflammation in patients with low‐risk PCa.

PLR is another inflammation marker that is considered to be as important as NLR. According to the results of a meta‐analysis investigating the relationship between PLR and PCa, high PLR was associated with poor disease‐free and overall survivals in patients independent of ethnicity, tumour stage and cut‐off value [29, 30]. In a study conducted on patients with PCa receiving androgen deprivation treatment, PLR was found to be a parameter that affected prognosis [31]. In a study involving patients with PCa under AS, it was stated that both NLR and PLR did not predict upgrade and treatment [32]. In the present study, PLR was found to be one of the most effective parameters in predicting the need for curative treatment in patients under AS.

In patients with PCa, another biomarker whose popularity has been increasing in recent years is SII. SII is generally investigated in patients with castration‐resistant PCa. Recently, it has also been investigated in patients with PCa who are at different disease stages. In a study wherein patients were evaluated before the first biopsy, SII was found to be ineffective in differentiating clinically important PCa from unimportant or benign prostate enlargement [33]. In another study including 80 patients with metastatic castration‐resistant PCa, NLR > 3, PLR > 150 and/or SII > 535,000 was associated with poor prognosis [34]. In another study conducted in patients with metastatic castration‐resistant PCa treated with docetaxel, SII, albumin and fibrinogen levels were reported as independent risk factors predicting poor prognosis [35]. Similarly, in a study by Fan et al., SII was defined as an independent negative factor affecting overall survival [36]. There is no study in the literature investigating the use of SII in predicting treatment need in patients under AS. Therefore, the results of the present study are therefore extremely valuable. SII will take its place in the literature as a valuable biomarker, like NLR or PLR, in patients followed with AS.

The limitations of this study include retrospective evaluation, the inclusion of a relatively low number of patients and lack of evaluation of inflammatory biomarkers at the tissue level using molecular biology approaches. However, the absence of a biomarker that predicts the need for curative treatment in the early period of AS highlights the importance of this study on these inflammation markers.

## 5 Conclusion

AS is a reliable method recommended in PCa guidelines. NLR, PLR and SII evaluated before making the decision to perform AS, which is important in the management of patients with PCa, are useful biomarkers for predicting the need for curative treatment in the early period. Thus, while additional costs and morbidity caused by repeated follow‐ups are reduced, the possibility of requiring curative treatment will be preserved. In the future, determining the optimal cut‐off values of these biomarkers is of utmost importance so that they can be used as one of the criteria for selecting patients for AS.

## Disclosure

The authors have nothing to report.

## Ethics Statement

This descriptive retrospective study was approved by the local ethics committee (approval no. 2021/14/31) and conducted in accordance with the World Medical Association's Declaration of Helsinki, ‘Ethical Principles for Medical Research Involving Human Subjects’.

## Conflicts of Interest

The authors declare no conflicts of interest.

## Data Availability

Research data are not shared.
